# Activation of autophagy triggers mitochondrial loss and changes acetylation profile relevant for mechanotransduction in bladder cancer cells

**DOI:** 10.1007/s00204-022-03375-2

**Published:** 2022-10-10

**Authors:** Maximilian Jobst, Endre Kiss, Christopher Gerner, Doris Marko, Giorgia Del Favero

**Affiliations:** 1 Department of Food Chemistry and Toxicology, Faculty of Chemistry, University of Vienna, Währingerstr. 38-40, 1090 Vienna, Austria; 2 Core Facility Multimodal, Imaging, Faculty of Chemistry, University of Vienna, Währingerstr. 38-40, 1090 Vienna, Austria; 3 Department of Analytical Chemistry, Faculty of Chemistry, University of Vienna, Währingerstr. 38-40, 1090 Vienna, Austria; 4 Joint Metabolome Facility, University of Vienna and Medical University of Vienna, Vienna, Austria

**Keywords:** T24 bladder cancer cells, Mitophagy, Rapamycin, Migration, Acetylation, Shear stress (fluid)

## Abstract

Bladder cells are constantly exposed to multiple xenobiotics and bioactive metabolites. In addition to this challenging chemical environment, they are also exposed to shear stress originating from urine and interstitial fluids. Hence, physiological function of bladder cells relies on a high biochemical and biomechanical adaptive competence, which, in turn, is largely supported via autophagy-related mechanisms. As a negative side of this plasticity, bladder cancer cells are known to adapt readily to chemotherapeutic programs. At the molecular level, autophagy was described to support resistance against pharmacological treatments and to contribute to the maintenance of cell structure and metabolic competence. In this study, we enhanced autophagy with rapamycin (1–100 nM) and assessed its effects on the motility of bladder cells, as well as the capability to respond to shear stress. We observed that rapamycin reduced cell migration and the mechanical-induced translocation potential of Krüppel-like transcription factor 2 (KLF2). These effects were accompanied by a rearrangement of cytoskeletal elements and mitochondrial loss. In parallel, intracellular acetylation levels were decreased. Mechanistically, inhibition of the NAD + -dependent deacetylase sirtuin-1 (SIRT1) with nicotinamide (NAM; 0.1–5 mM) restored acetylation levels hampered by rapamycin and cell motility. Taken together, we described the effects of rapamycin on cytoskeletal elements crucial for mechanotransduction and the dependency of these changes on the mitochondrial turnover caused by autophagy activation. Additionally, we could show that targeted metabolic intervention could revert the outcome of autophagy activation, reinforcing the idea that bladder cells can easily adapt to multiple xenobiotics and circumvent in this way the effects of single chemicals.

**Supplementary Information:**

The online version contains supplementary material available at 10.1007/s00204-022-03375-2.

## Introduction

Bladder cancer is the most common type of cancer in the urinary tract and the 4th most common cancer overall for men (6% of all new cases and 4% of cancer related deaths). (Siegel et al. 2022) Treatment is highly invasive with radical cystectomy and platinum based combination chemotherapy being the gold standard for muscle invasive bladder cancer (Gore et al. 2010; Fletcher et al. 2011; Tan et al. 2015). If the disease is detected at an early stage, trans urothelial resection of the bladder tumor (TURBT) is often performed. The main advantage of TURBT is that it does not interfere with bladder function; however, this comes at the price of higher rates of tumor recurrence (Manoharan 2011). Prevention and improved treatment lowered the lethality and occurrence of many of the most common cancer types (i.e., colon and lung); however, such success stories are comparatively limited for bladder cancer (Siegel et al. 2022). Therefore, understanding bladder cancer pathophysiology and elucidating the underlying molecular mechanisms is of utmost importance to develop new treatment options.

Cell lines have been a great tool for the development of new drugs and to enhance our understanding of pathophysiological processes (Shoemaker 2006; Barretina et al. 2012). However, more work is needed to fully model the in vivo conditions in our in vitro assays. In vivo bladder cells are confronted by a challenging environment: they are exposed to a combination of chemical stressors including bioactive metabolites and xenobiotics. These mixtures originate from dietary and environmental exposure to chemicals or as the result of pharmacological treatment. Additionally, physical cues shape the physiology of the bladder, including deformation which derives from shear stress by interstitial fluids and urine, as well as from expansion and contraction of the organ. To withstand this complex environment, bladder cells must be able to cope and adapt to these stressors with a sophisticated mechanosensory apparatus (Dalghi et al. 2019). This extends also to pathological conditions, and, on these molecular premises, it is possible to hypothesize that the aggressiveness of bladder tumors could be traced back to the necessary adaptive plasticity of the bladder cells ancestries.

Looking for pathways potentially accounting for the adaptive capacity of bladder cancer cells, the role of autophagy is emerging (Chandrasekar and Evans 2016). Hence, autophagy can be involved in response to toxicants and its modulators can affect the activity profile of noxious chemicals (Bolt and Klimecki 2012; Tan et al. 2020). Additionally, autophagy can influence the outcome of therapeutic agents and can also complement therapy to counteract autophagy mediated chemoresistance (Li et al. 2019a). Complementary to pharmaco-toxicological considerations, autophagy can tune the response of bladder cells to shear stress (Del Favero et al. 2021b) and this is in line with the emerging concept that an intensive crosstalk between physical stress and autophagy could support cancer progression (Dupont and Codogno 2016). Through autophagy, cells break down and recycle cellular components in a controlled manner, this can range from single proteins to complete organelles. Autophagy can occur unselectively in times of nutrient deficiency, but also selectively to control quality, number, and the overall performance of organelles (Aman et al. 2021). For example, in a process termed mitophagy, old or damaged mitochondria are recycled for the sake of newer ones to optimize cellular metabolism (Youle and Narendra 2011; Chen et al. 2020; Del Favero et al. 2020a). Autophagy plays a critical role in cancer development and progression; on the one hand, it can prevent malignant transformation of cells by removing misfolded proteins and damaged organelles; however, it also increases survival of already existing cancer cells, fostering chemoresistance and preventing of apoptosis (Rubinsztein et al. 2012; Mulcahy Levy and Thorburn 2020; Hernández-Cáceres et al. 2021). Hence, understanding the mechanisms regulating autophagy and stress response capacity of cancer cells might play a significant role in understanding the impact of long term exposure to xenobiotics in sub-cytotoxic concentrations. Crucially, the contribution of mechanotransduction is emerging as a complementary element potentially affecting cellular response to xenobiotics and pharmacological treatment. In this light, more and more studies are contributing to establish a link between mechanical stress, autophagy and pharmacological resistance. To name some examples, fluid shear stress induces autophagy by activation of Rho GTPases Rac1, RhoA, and Cdc42 (Wang et al. 2018; Yan et al. 2019). Sensing of the extracellular matrix through integrin-mediated adhesion regulates autophagy and has been linked to increased chemoresistance and survival of detachment-induced cell death (anoikis) (Lock and Debnath 2008; Vlahakis and Debnath 2017; Anlaş and Nelson 2020). In SKOV-3 ovarian cancer cells, application of shear stress in vitro triggered a complex post-translational protein modification signature which can be traced back to mTOR regulation, as well as to the regulation of proteins governing cell motility and morphology (Bileck et al. 2022).

Rapamycin, also known as sirolimus, is an established promotor of autophagy. The underlying mechanism is based on rapamycin’s inhibition of mTORC1 kinase activity; this in turn blocks the ability to control ULK1/2 phosphorylation, which hampers autophagosome formation in normal conditions (Noda and Ohsumi 1998; Kim et al. 2002; Jung et al. 2010). We previously demonstrated that xenobiotics-induced modulation of autophagic competence shapes the response profile of T24 bladder cells to shear stress (Del Favero et al. 2021b). In this study we aimed to further elucidate the intracellular cascades triggered by the autophagy activator rapamycin and how this might reflect on cell biomechanical compliance. Hence, the effects of rapamycin on cell motility and on the intracellular architecture including cytoskeletal elements and mitochondria were taken as a starting point. Additionally, translocation potential of transcription factor krüppel-like factor 2 (KLF2) was evaluated; since rapamycin was previously shown to regulate the expression and activity of KLF2 (Ma et al. 2012), which in turn was described to regulate mitophagy (Maity et al. 2020) and to be mechanosensitive (Parmar et al. 2006). Overall, we aimed to elucidate systematically the effects of autophagy modulation on mechanotransduction and use this as an endpoint to evaluate the efficacy of chemical and pharmacological intervention in bladder cancer cells.

## Materials and methods

### Chemicals and materials

InSolution Rapamycin [Calbiochem 553,211; 1–100 nM (Foster and Toschi 2009)], Bafilomycin A1 [bafilomycin, SML-1661; 10 nM (Redmann et al. 2016)] and Nicotinamide [N0636; 0.1–5 mM Avalos et al. 2005; Hwang and Song 2017)], Yoda1 [REF: SML 1558; 5 µM (Davies et al. 2019)], Filipin complex ready-made solution (REF: SAE0088) were purchased from Sigma-Aldrich (USA). The compounds were dissolved in DMSO or water according to the technical specification of the suppliers. Solvent controls were matched to represent the same solvent concentrations of the treatments which were obtained by diluting stock solutions 1:1000. For the immunofluorescence studies, the following antibodies were used: anti-acetylated Lysine Monoclonal Antibody (1C6) (MA1-2021, Invitrogen), anti-Caveolin-1 (ab192452, Abcam, dil 1:500), anti-Integrin beta 1 (ab30394, Abcam, dil 1:500), anti-PMP70 (PA1-650, Invitrogen dil 1:500), anti-CLUH (PA5-65,101, Invitrogen, dil 1:500), anti-KLF2 (ab203591, Abcam, dil 1:600), anti-TOM20 (sc-17764, Santa Cruz Biotechnology, dil 1:500), Alexa Fluor® 647 donkey anti-mouse IgG (H + L) (A31571, Invitrogen, dil. 1:1000), Alexa Fluor® 647 donkey anti-goat IgG (H + L) (A21447, Invitrogen, dil. 1:1000), Alexa Fluor® 568 donkey anti-rabbit IgG (H + L) (A10042, Invitrogen, dil. 1:1000), Oregon Green® 488 Phalloidin (O7466, Invitrogen, dil. 1:1000).

### Cell culture

The urinary bladder carcinoma cell line T24 (ATCC® HTB4™) was purchased from ATCC. T24 cultures were performed according to the specification of the supplier using McCoys 5A medium (Gibco REF 22330-021) supplemented with 10% (v/v) fetal calf serum 1% (v/v) Penicillin/Streptomycin in TC-Flasks T75 (Sarstedt REF 83.3911.002). For cultivation and all incubations humidified incubators were used at 37 °C and 5% CO_2_. Cells were used for assays at confluence between 80 and 90%.

### Migration assay

For migration assay, 140,000 T24 cells were seeded with 2 ml cell culture medium in 35 mm 6-well plates (Sarstedt REF 83.3920.005). The assays were performed 48 h after the seeding, at this point almost full confluence was achieved. Scratching was done using a 200 µL pipette tip, followed by a washing step with medium. The tip was changed every 3 wells. After addition of 2 ml treatment solution, pictures were taken on Lionheart FX automated microscope using GEN5 Microplate Reader and Imager Software Version 3.05 for imaging and quantification both from BioTek Instruments Inc. (Vermont, USA). After 24 h incubation at 37 °C and 5% CO_2,_ a secondary set of pictures was taken at the same coordinates. Quantification of the healed area was performed using ImageJ 1.53a software. 3 experimental replicates were performed and at least 3 optical fields were evaluated, resulting in at least 9 optical fields per condition.

### Biophysical stimulation

To create shear stress in vitro, two technical approaches were used. For the migration assays we used the MK3 control orbital shaker (IKA, Staufen, Germany) placed inside our incubator. We exposed the cells to 250 RPM for 24 h corresponding to approximately 2.8 dyn/cm^2^ as previously described (Warboys et al. 2019; Bileck et al. 2022). For the KLF2 translocation experiment, shear stress was generated using the Ibidi Pump System Quad (Ibidi GmbH, Martinsried, Germany), cells were seeded in µ-Slide I^0.4^ Luer Collagen IV (REF: 80,172, Ibidi GmbH, Gräfelfing, Germany) and a shear stimulus was applied corresponding to 2.7 dyn/cm^2^ (Del Favero et al. 2021b), which is in the range of the physiological shear stress experienced by this cell type (Lee et al. 2018). Experiments were repeated in at least 3 independent cell preparations (biological replicates).

### Cholesterol quantification

Cell membrane cholesterol was detected by filipin staining as described previously (Rebhahn et al. 2022). Briefly, cells were fixed with 1% formaldehyde in DPBS at room temperature for 15 min, washed in DPBS, blocked with Glycin/DPBS then washed again. Cells were incubated in the presence of filipin solution (50 µg/ml in DPBS) at room temperature in dark for 60 min. After a subsequent washing step, cells were mounted in ROTIMount FluorCare mounting medium (REF: HP19.1, Carl Roth, Germany) and imaged using a Zeiss LSM710 laser scanning confocal microscope (ELYRA PS.1 system) with a 63X/1.46 Plan-Apochromat oil immersion objective (Zeiss Microscopy GmbH, Germany).

### Membrane fluidity assay

Evaluation of membrane fluidity was performed as previously described (Zhang et al. 2011; Del Favero et al. 2021a; Rebhahn et al. 2022). Briefly, cells were seeded in a black 96-well plate with clear bottom and incubated for 24 h with rapamycin 1–100 nM. The day of the measurements, cells were loaded for 1 h with 1-pyrendecanoic acid (10 µM, PDA, Thermo Fisher Scientific, Waltham, MA, USA). Fluorescence emission signals for PDA monomers (375 nm; *I*_m_) and excimers (470 nm; *I*_e_) were measured after excitation (344 nm) with a Cytation3 Imaging Multi-Mode Reader (BioTek, Winooski, VT, USA). Membrane fluidity was expressed as ratio *I*_e_/ *I*_m_ and compared to solvent controls. Experiments are mean of 3 independent biological replicates performed at least in technical quadruplicates.

### Acetylation assay

For acetylation assay 20,000 T24 cells were seeded with 300 µl cell culture medium in 48-well plates (Sarstedt REF 83.3920.005). After 24 h cells were incubated with 300 µL treatment solution for 24 h at 37 °C and 5% CO_2_. After removal of the incubation medium and a PBS-A washing step, fixation was performed using 3.5% formaldehyde in PBS-A for 10 min. After another PBS-A washing step, formaldehyde was quenched using a Glycine/PBS-A solution. Following a third PBS-A washing step, plates were stored overnight at + 4 °C. Fixed cells were permeabilized with 0.2% Triton ×-100 for 15 min and were blocked using 20% normal goat serum in PBS-A. Incubation with respective primary antibody solution was performed for 2 h. Cells were washed three times with washing buffer (0.05% Triton-× in PBS-A) and twice with PBS-A. Secondary antibodies and phalloidin incubation was performed following the same procedure as the primary antibody. 70 µL mounting medium (ROTH Roti®Mount FluorCare DAPI ArtNr: HP 20.1) was added to each well. Imaging of acetylated lysine was performed with Lionheart FX Automated microscope from BioTek (Vermont, USA). Image acquisition and quantification was performed using GEN5 Microplate Reader and Imager Software Version 3.05 from BioTek (Vermont, USA). The images were composed from the channels: DAPI [377,447 nm], CY5 [628,685 nm], and GFP [469,525 nm]. To quantify acetylation levels mean fluorescence intensity was measured for each cell considering nuclear and cytoplasmic area respectively.

### Immunofluorescence

Immunofluorescence experiments were performed according to the same protocol as described for acetylation assay as and adapting previously described workflows for PMP70 (Niederstaetter et al. 2021), Caveolin-1 (Del Favero et al. 2020b), Integrin beta 1 (Groestlinger et al. 2022) and TOM20 (Del Favero et al. 2021a). The experiments were performed in µ-Slide 8 well Collagen IV (REF: 80,822, Ibidi GmbH, Gräfelfing, Germany), using the same cell number and volumes as the 48-well plates and blocking of the unspecific binding sites was obtained with 2% donkey serum for one hour. Images were obtained using Zeiss LSM710 laser scanning confocal microscope (ELYRA PS.1 system) with a 63X/1.46 Plan-Apochromat oil immersion objective (Zeiss Microscopy GmbH, Germany). The software ZEN 2012 Black Edition (Zeiss Microscopy GmbH, Germany) was used for analysis and quantification of the images. According to the datasets, images were quantified taking region of interest (ROIs, mean fluorescence relative units r.u.) or total cell area as reference (% area of the signal of interest/total cell area). For all the experiments, measurements were performed on at least 3 independent cell preparations (biological replicates) and quantifying *n* > 50 ROIs/cells out of minimum 9 randomly chosen optical fields.

### Cell viability and proliferation assays

Cell count was determined by quantifying nuclei per images, following DAPI staining. This task was performed automatically using Lionheart FX automated microscope using GEN5 Microplate Reader and Imager Software Version 3.05 for imaging and quantification, both from BioTek Instruments Inc. (Vermont, USA). Additionally, we determined cell viability via biomass performing a crystal violet assay using a protocol previously described (Del Favero et al. 2021b). Briefly, cells were rinsed with warm DPBS before fixation with cold ethanol (99%). Staining was performed for 5 min using crystal violet solution (0.1%). Cells were washed 4 times using autoclaved water. Cells were lysed for 10 min on an orbital shaker (500U/min) using 99% EtOH and acetic acid (1%), before measuring absorbance [595 nm; Cytation 3 multi-mode plate reader (BioTek Instruments, Winooski, VT, United States)]. Three independent cell preparations were prepared and three technical replicates were measured.

### Statistical evaluation

Statistical evaluation and creation of all graphs was performed using OriginPro, Version 2021. OriginLab Corporation, Northampton, MA, USA. Student’s *t*-test (*n* > 40), Mann–Whitney Test (*n* < 40) and ANOVA tests (*n* ≥ 3, dose dependent datasets) were applied for the comparison of groups of data and *p* values smaller than 0.05 were considered statistically significant. At least three independent cell preparations (biological replicates) were used for each experimental workflow.

## Results

### Effects of rapamycin on cell biomechanical compliance

**Fig. 1:**
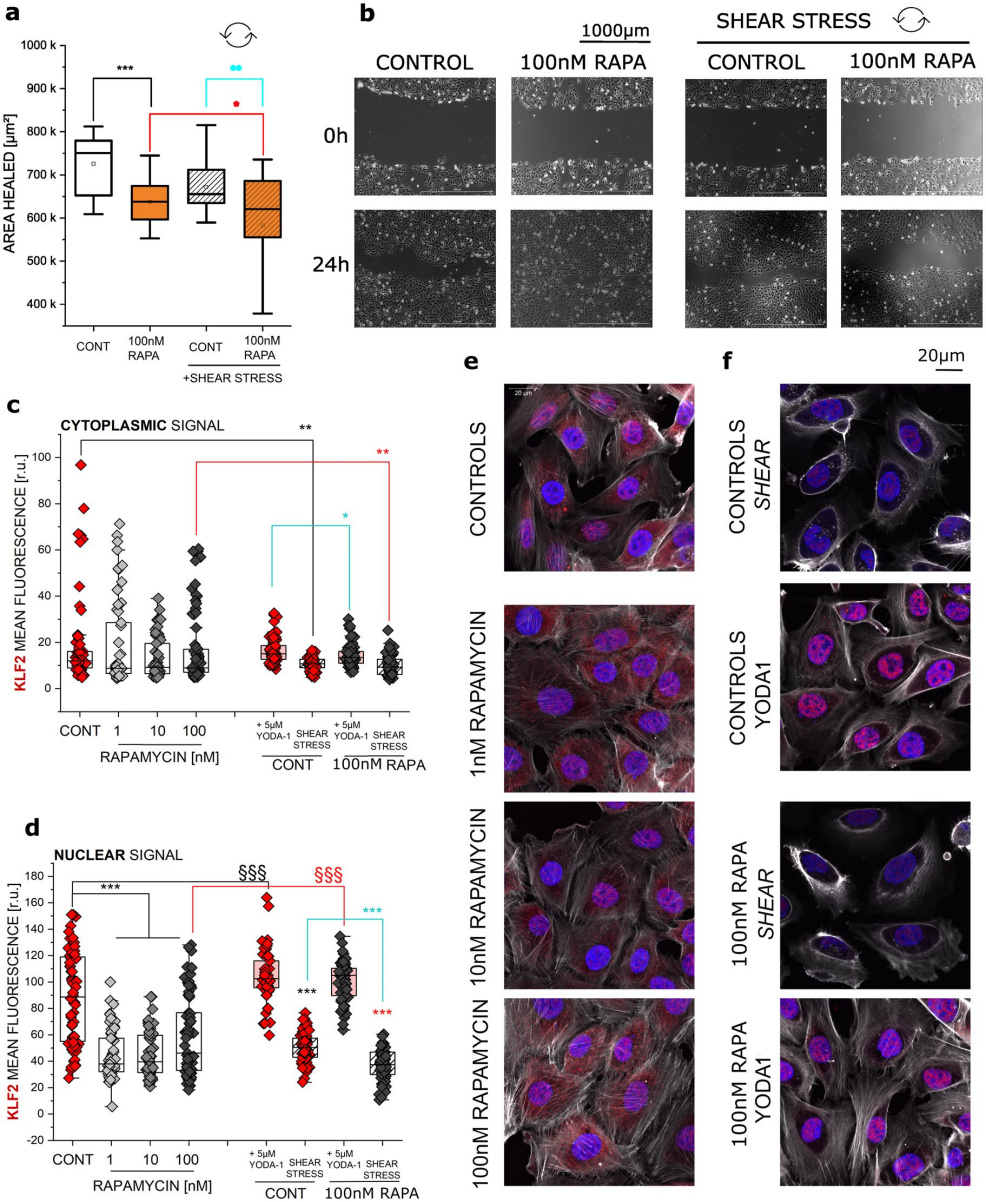
Changes in migration of T24 cells when exposed to rapamycin (RAPA) and/or shear stress **a** quantification of the gap-closure assay, results are given as area healed [µm^2^], calculated as the difference between the initial area of the scratch minus the area at the same coordinates after 24 h incubation (*n* = 12 optical fields); *indicates a significant difference at Mann–Whitney Test (*p < 0.05; **p < 0.01; ***p < 0.001); **b** Representative phase contrast images at both *t* = 0 and *t* = 24 h, shear stress incubation is indicated by curved arrows (scale bar: 1000 µm); **c**, **d** Quantification of the mean fluorescence intensity of KLF2 in cytoplasmic **c **and nuclear **d** area taken from the evaluation of *n* > 50 cells; n.s. indicates no statistical significant difference to control according to the Student's *t*-test (*p* > 0.05) * indicates a significant difference in comparison to controls and § among treatments (*§ *p* < 0.05; **§§ *p* < 0.01; ***§§§ *p* < 0.001); **e** Representative images of actin and KLF2 following 24 h incubation with RAPA (1–10-100 nM) and control cells. **f** Images depicting shear stress incubation (*SHEAR*; 2.7 dyn/cm^2^) or YODA1 (5 µM) treatment respectively; DAPI stained nuclei are depicted in blue, actin in white, KLF2 in red (scale bar: 20 µm)

Starvation induced autophagy was previously reported to increase both migration and invasiveness of bladder cancer cells (Tong et al. 2019). Hence, as a first step in the assessment of the effects of autophagy activator rapamycin on T24 cells motility, migration experiments were performed. For this purpose, we measured cell migration in a gap-closure assay, during 24 h in the presence or absence of 100 nM rapamycin treatment. Additionally, given the fact that bladder cells are constantly exposed to fluid shear stress we implemented in our experimental setup respective biomechanical stimulation to gain insights on the physical stress resistance of the T24 cell line. Differently to serum starvation induced autophagy (Supplementary Fig. 1), incubation with rapamycin decreased the migration capacity of treated cells when compared to controls. Application of fluid shear stress stimulation protocol slowed migration compared to static conditions and the co-application of rapamycin and shear stress hampered significantly the motility of bladder cells (Fig. 1a and b). To verify if this response behavior could be observed also for other experimental endpoints, we decided to determine the localization of KLF2 transcription factor (Fig. 1c–f). Krüppel-like factor 2 (KLF2), is described as a mechanosensitive transcription factor (Niu et al. 2019; Dabravolski et al. 2022), hence can be activated by physical triggers, and was recently shown to inhibit cell migration in various forms of cancers (Wang et al. 2019; Lin et al. 2019). Moreover, it is described as a molecular effector of the autophagic cascade (Guixé-Muntet et al. 2017; Laha et al. 2019). We measured KLF2 in the cytosolic (Fig. 1c) as well as in the nuclear region of T24 cells (Fig. 1d) to assess eventual translocation patterns, as previously described for other transcription factors like Nrf2 (Jarolim et al. 2017). Despite signal fluctuation, no significant changes were measured in the cytoplasmic region (Fig. 1c) after 24 h static incubation with rapamycin 1–100 nM. However, we observed that incubation with rapamycin significantly lowered KLF2 levels in the nucleus of T24 cells (Fig. 1d). 3 h shear stress stimulation protocol was applied to evaluate potential effects on the mechanically induced translocation potential of the transcription factor. In control conditions, mechanical stimulation protocol reduced KLF2 detection in both nuclear and cytoplasmic compartments. Following the response profile of the migration assays, exposure to shear stress after rapamycin treatment lowered the detection of KLF2 even further, compared to static conditions and in comparison to controls (Fig. 1c–f). Overall, exposure to rapamycin seemed to exacerbate the effects of physical stimulation. Building on this, our hypothesis was that the compound could modify cell structure and weaken cell cytoskeleton, as suggested also by the rearrangement of actin visible in the immunofluorescence images (Fig. 1e and f). To verify the dependency of the translocation results from the cytoskeletal integrity, experiments were performed in presence of YODA1. YODA1 is a chemical agonist of mechanosensitive PIEZO1 ion channels, which was previously used to mimic the effects of fluid shear stress without the application of physical forces on the cells and, consequently, requiring a reduced involvement of the cytoskeletal system (Davies et al. 2019). 3 h incubation with YODA1 increased KLF2 signal in the nucleus in both control and rapamycin treated cells, fitting the idea that the opening of the PIEZO1 eased the translocation of KLF2 (Fig. 1d). Coherently with a comparable intensity of actin cytoskeleton staining (Fig. 1e–f), the translocation potential measurable in control cells and in rapamycin treated cells appeared very similar. Only a moderate decrease of KLF2 in the cytoplasmic compartment upon incubation with rapamycin and YODA1 in comparison to YODA1 alone could be observed (Fig. 1c).

### Effect of rapamycin on cytoskeletal proteins

**Fig. 2:**
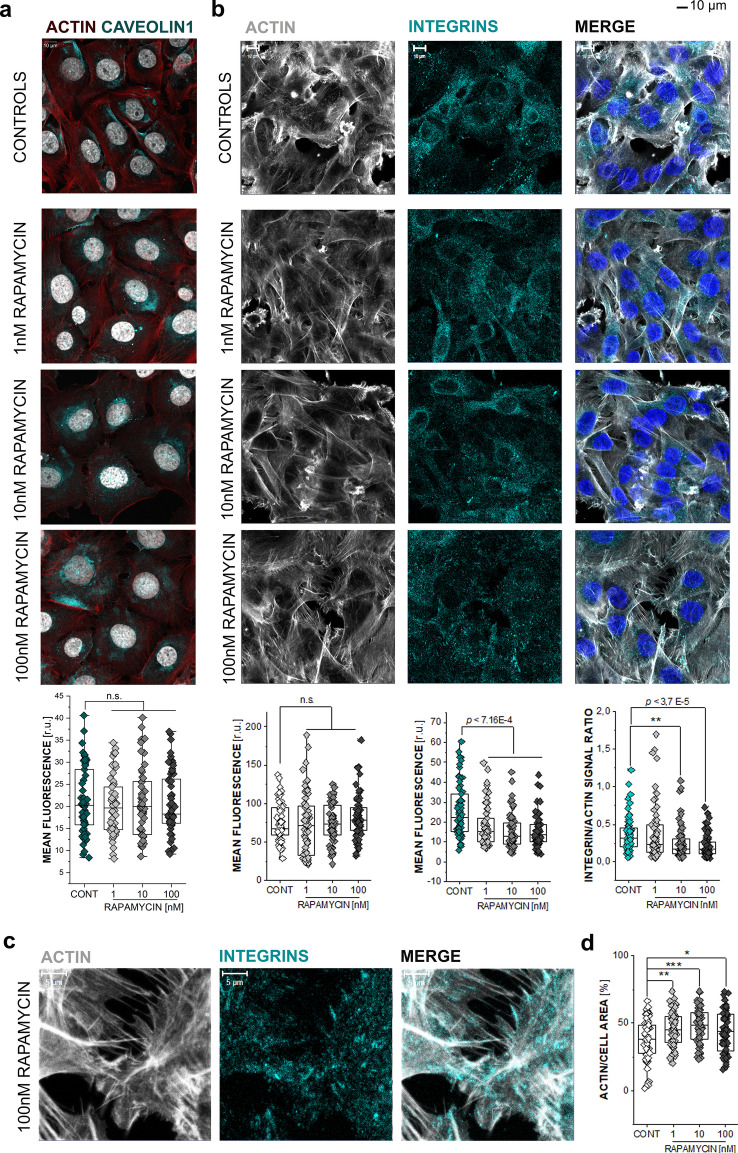
Cytoskeletal response to rapamycin. **a** Representative images of actin and caveolin1 and corresponding quantification, following 24 h incubation with rapamycin (RAPA) and control cells. Data is given as mean fluorescence per ROIs, taken from *n* > 50 cells; n.s. indicates no significant difference (*p* > 0.05) for Student’s *t*-test; Actin is depicted in red, caveolin in teal and nuclei in white; (scale bar: 10 µm). **b** Comparisons of actin and integrin levels in T24 cells following 24 h RAPA treatment, representative images (scale bar: 10 µm), and quantification of *n* > 60 ROIs, results are given as mean fluorescence intensity, n.s. indicates no significant difference (*p* > 0.05) for Student's *t*-test statistical significance is shown via ** (*p* < 0.01), *** (*p* < 0.001) by listing the corresponding *p* value; Actin is depicted in white, integrins in teal and nuclei in blue. **c** Representative image of T24 cells after 100 nM RAPA treatment for 24 h, with increased magnification (scale bar: 5 µm); actin is shown as white integrins in teal. **d** Quantification of the percentage of cell area covered by actin signal *n* > 60 cells statistical significance is shown via Student’s *t*-test: * (*p* < 0.05), ** (*p* < 0.01), *** (*p* < 0.001)

Building on the outcome of the migration and KLF2 translocation assays, it was started to explore if incubation with rapamycin could be associated to changes in the cytoskeleton of T24 bladder cells. For this, key cytoskeletal proteins like actin, caveolin1 (CAV1) and integrin β1 were investigated, which are essential components of the cells mechanosensory apparatus (Zemljič Jokhadar et al. 2013). Incubation with rapamycin did not modify the distribution and detection of CAV1 (Fig. 2a). Integrin β1 signal was reduced after rapamycin treatment (Fig. 2b) and the immunolocalization of the adhesion protein showed redistribution along the actin cytoskeleton especially in the cell periphery (Fig. 2c). In addition, incubation with rapamycin resulted in a concentration dependent decrease of the ratio between the integrins and actin. In parallel, mean actin fluorescence intensity did not change significantly (Fig. 2b). However, the actin signal distribution within the cells (expressed as actin/cell area ratio) increased after treatment in a concentration dependent fashion (Fig. 2d). Taken together, these findings show that rapamycin modulates the cytoskeleton of T24 bladder cells, not necessarily by lowering the overall actin levels inside the cell, but rather by affecting its spatial distribution.

### Effect of rapamycin on cholesterol and PMP70

**Fig. 3:**
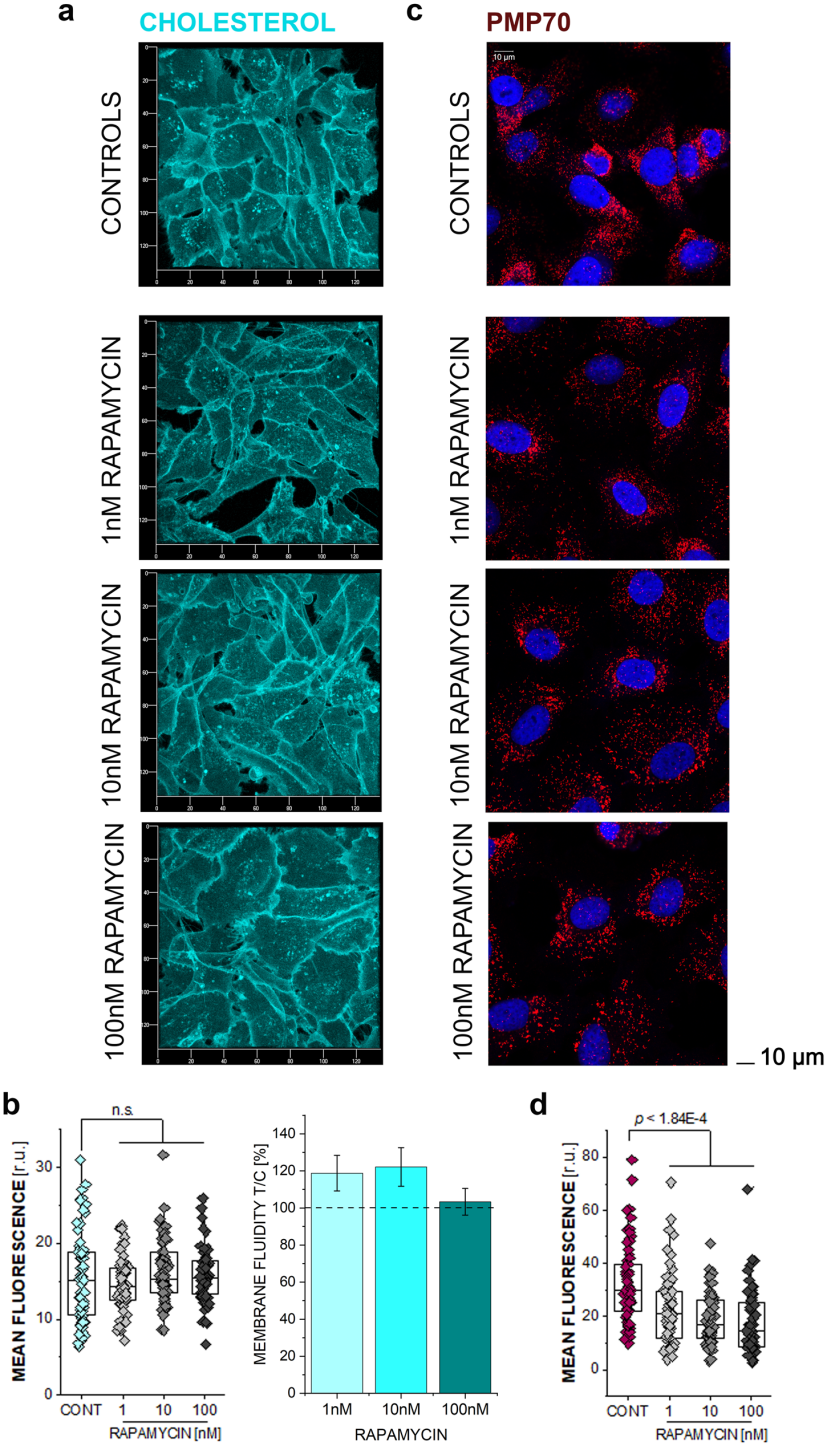
Cholesterol and PMP70 response after 24 h treatment with increasing rapamycin (RAPA) concentrations **a** representative images of cholesterol staining in control cells and after incubation with rapamycin; cholesterol in blue (x/y axis in 20 µm segmentation) **b** quantification of the mean fluorescence intensity of cholesterol (*n* > 60 ROIs) and membrane fluidity (*n* = 3), taken from the evaluation of 3 independent experiments, n.s. indicates no statistical significant difference to control according to the Student’s and ANOVA tests (*p* > 0.05), **c** PMP70 response in control cells and after incubation with RAPA; PMP70 in red, nuclei in blue (scale bar: 10 µm) **d** quantification of the mean fluorescence intensity of PMP70, from *n* > 60 cells. Statistically significant differences were calculated via Student’s *t*-test (*p* < 1.84E-4)

To start shedding light on the mechanisms potentially sustaining the changes in mechanical behavior of bladder cells after exposure to rapamycin, experiments were performed to assess a potential involvement of the cell membrane. Indeed, for mechanical stress applied to the cells, the plasma membrane represents a primary integration site and alteration of its composition or biophysical properties can account for altered mechanotransduction with downstreaming involvement of the cytoskeleton (Ayee et al. 2017; Del Favero et al. 2018; Rebhahn et al. 2022). Moreover, membrane architecture is crucial to host focal adhesions and integrins, which appeared modified by the incubation with rapamycin. 24 h incubation with rapamycin had no effect on the distribution of membrane cholesterol and on morphology of T24 bladder cells (Fig. 3a). In agreement, no changes in the quantification of cholesterol binding dye filipin (mean fluorescence) or overall in the membrane fluidity could be observed (Fig. 3b). In parallel, the potential of rapamycin to affect intracellular organelles essential for lipid metabolism such as the peroxisomes was measured (Jo et al. 2020). In this case it was observed that the incubation with the autophagy activator rapamycin could trigger concentration-dependent decrease of the signal of PMP70, one of the major components of the peroxisomal membrane (Imanaka et al. 2000) (Fig. 3c and d).

### Effect of rapamycin on TOM20 and CLUH

**Fig. 4:**
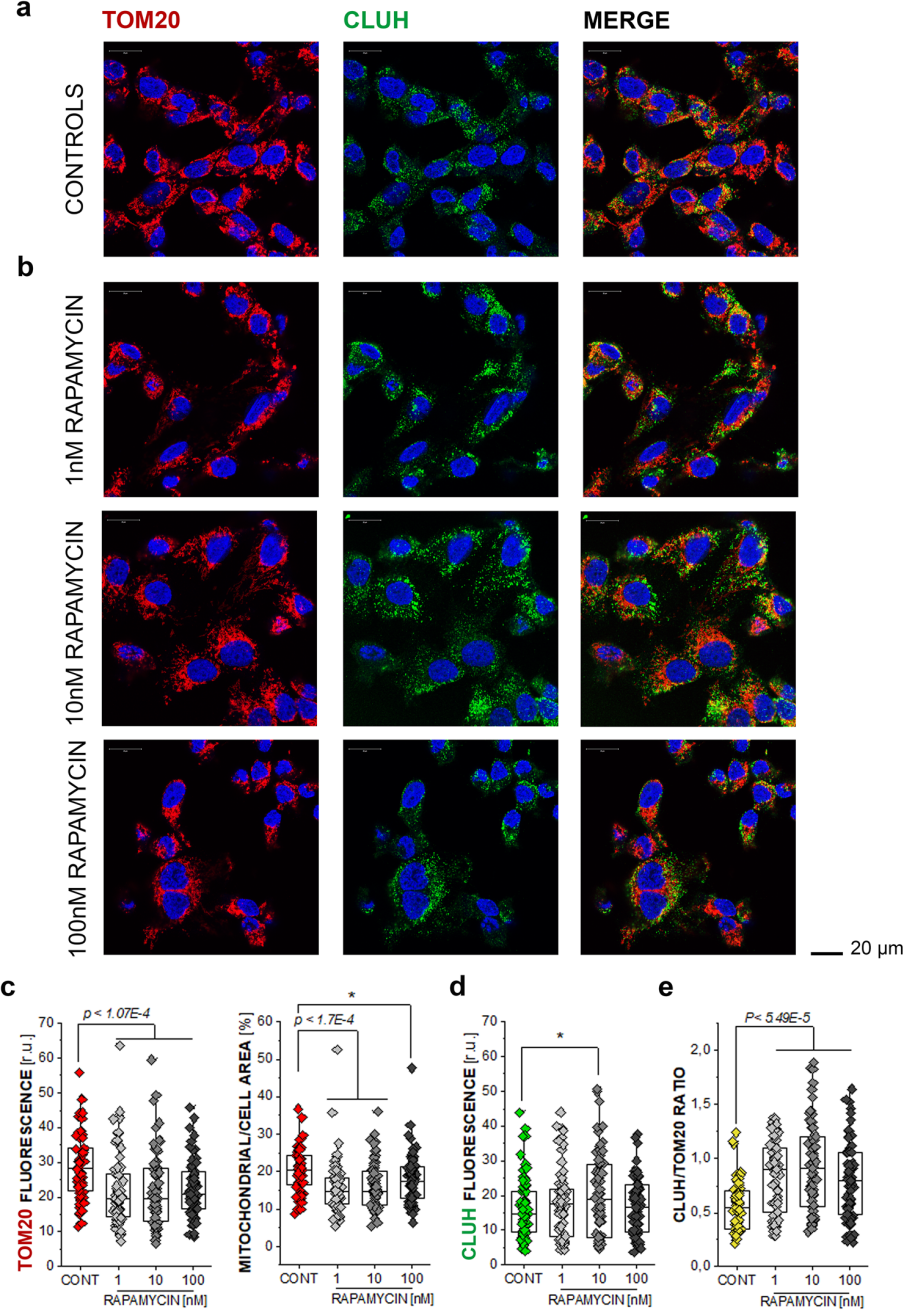
TOM20 and CLUH response after 24 h treatment with increasing rapamycin (RAPA) concentrations **a** representative images of control cells, as well as **b** cells after incubation with RAPA; TOM20 is depicted in red and CLUH in green, and nuclei in blue (scale bar: 20 µm) **c** quantification of the mean fluorescence intensity of TOM20 and the ratio of the mitochondrial area to the overall cell area, taken from the evaluation of *n* > 60 cells., * indicates statistical significant difference to control according to the Student’s *t*-test * (*p* < 0.05), or *** (*p* < 0.001) by listing the corresponding *p* value.  **d** CLUH response in control cells and after incubation with RAPA, quantification of the mean fluorescence intensity of CLUH, from *n* > 60 ROIs, from 3 independent experiments, statistical significant differences were calculated via Student’s *t*-test as (*p < 0.05), **e** quantification of the CLUH/TOM20 ratio in control cells and RAPA treated cells, from at least 9 random optical fields, from 3 independent experiments (*n* > 60 ROIs) statistical significant differences were calculated via Student’s *t*-test (*p* < 5.49E-5)

Since peroxisomes are tightly connected to the activity of the mitochondria (Wanders et al. 2016), we further analyzed the effect of rapamycin on the mitochondrial network. In line with the results of the PMP70, incubation of T24 cells with rapamycin severely impacted on the integrity of the mitochondrial network. Incubation with rapamycin significantly decreased the immunofluorescence localization of the mitochondrial import protein TOM20 (Fig. 4a and b). This was visible as a reduction of the mean fluorescence intensity of the TOM20 localization, as well as the cell area occupied by the mitochondrial signal, which is a measure of the spread of the mitochondrial network within the cell (Fig. 4c). In parallel, the protein CLUH, which regulates mitochondrial biogenesis (Gao et al. 2014) was increased in cells incubated with rapamycin 10 nM (Fig. 4d). Moreover, looking at the expression level of CLUH with the respect of TOM20, all cells treated with rapamycin resulted in an increased ratio in comparison to controls, as suggestive of a signature of increased mitophagy and organelle turnover (Fig. 4e).

### Effect of rapamycin on acetylation

**Fig. 5:**
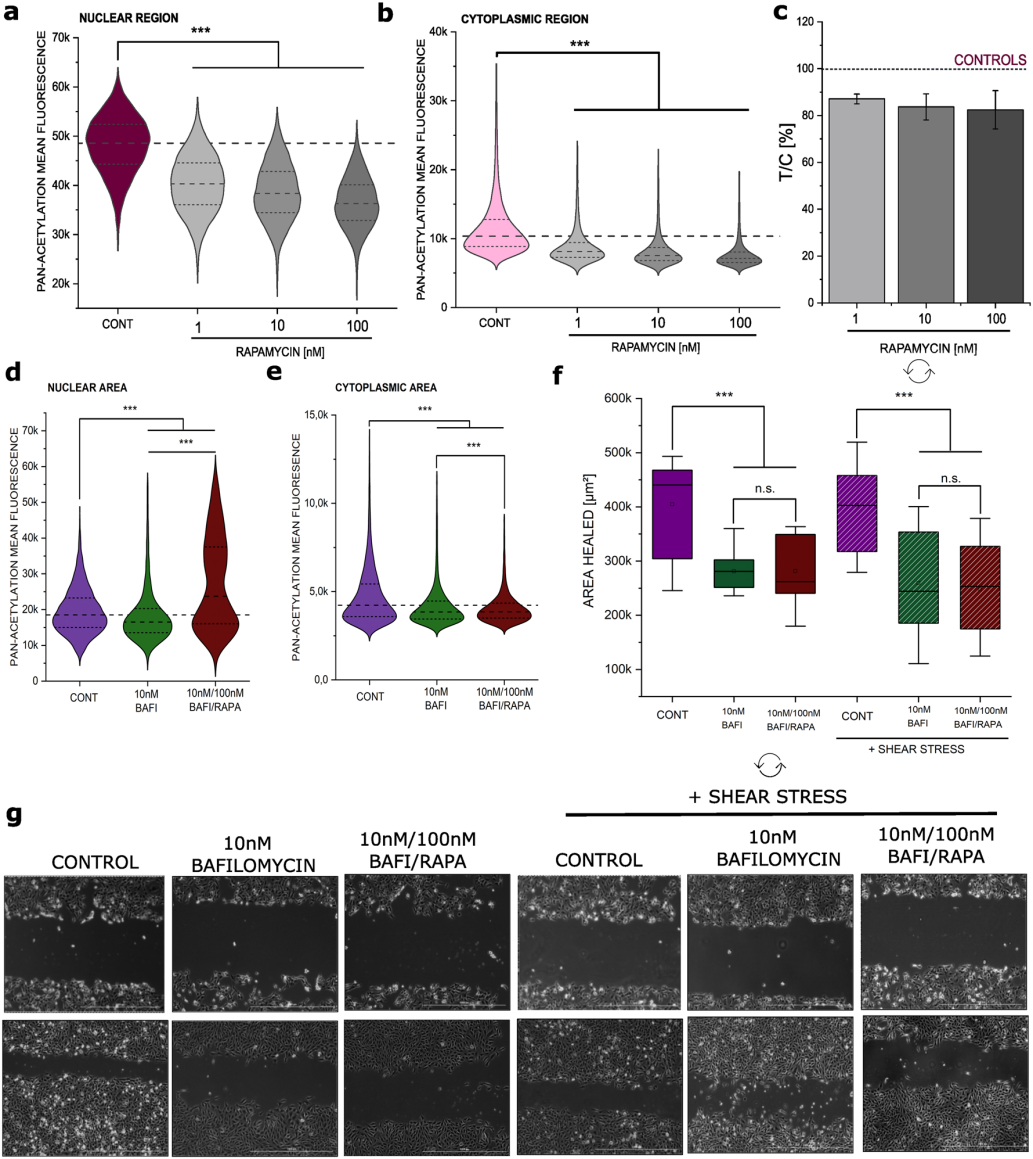
Changes in acetylation following 24 h rapamycin (RAPA) incubation in T24 cells. **a**, **b** Quantification of acetylated lysine in the nuclear (**a**) and cytoplasmic region of the cell (**b**). Quantification of cell viability (**c**) measured as cell biomass (crystal violet assay; *n* = 3). Changes in acetylation following 24 h incubation with bafilomycin (BAFI) and combination of BAFI and RAPA in T24 cells in the nuclear (**d**) and cytoplasmic region of the cell (**e**), results are given as mean fluorescent intensity per cell, *n* = 27 randomly selected optical field were quantified statistical significance is shown via Student's *t*-test: *** (*p* < 0.001). Quantification of the gap-closure assay (**f**), results are given area healed [µm^2^], calculated from the initial area of the wound minus the area at the same coordinates after 24 h incubation with BAFI or BAFI and RAPA in combination; * indicates a significant difference at Mann–Whitney test (***: *p* < 0.001); **g** Representative images at both *t* = 0 and *t* = 24 hours, taken with phase contrast (scale bar: 1000 µm)

Looking for pathways which could potentially account for the altered mechanotransduction of T24 cells in relation to mitochondrial loss, it was verified if the incubation with rapamycin could alter post-translational protein modifications (PTMs), and in particular acetylation. The predominant PTM in the cytoskeleton is acetylation, especially that of lysine (A et al. 2020). Furthermore, acetylation is a key regulator of autophagy, providing a possible link between autophagy modulation and the observed cytoskeletal changes (Bánréti et al. 2013). Rapamycin induced a concentration dependent decrease in the detection of acetylated lysine in both the nuclear and in the cytosolic region of the cells (Fig. 5a, b). In agreement with the morphological changes observed in the cytoskeleton (Fig. 2) rapamycin incubations decreased cell count/optical field in a concentration dependent manner (Supplementary Fig. 2); this appeared most likely associated with a change in cell morphology and increased cell spread, rather than a cytotoxicity event, hence measurement of cell biomass with the crystal violet assay returned a decrease in comparison to control cells, which stabilized between 80 and 90% in comparison to solvent controls (Fig. 5c). To verify if the decreased acetylation and cell migration could be rescued by autophagy inhibition, experiments were performed with the inhibitor bafilomycin (10 nM). Even if the treatment with bafilomycin reduced the acetylation in the nuclear compartment, the combination with rapamycin increased signal to values superior to those of the controls (Fig. 5d). However, in the cytoplasmic compartment this effect was not visible, and both bafilomycin and bafilomycin–rapamycin treatments reduced the acetylation in this area (Fig. 5e). In line to the postulated effects of acetylation on the cytoskeleton, obviously more prominent in the cytoplasmic compartment, co-incubation with rapamycin (100 nM) and bafilomycin (10 nM) failed to restore motility in T24 cells (Fig. 5f–g).

### Effect of nicotinamide on acetylation and migration in combination with rapamycin

**Fig. 6:**
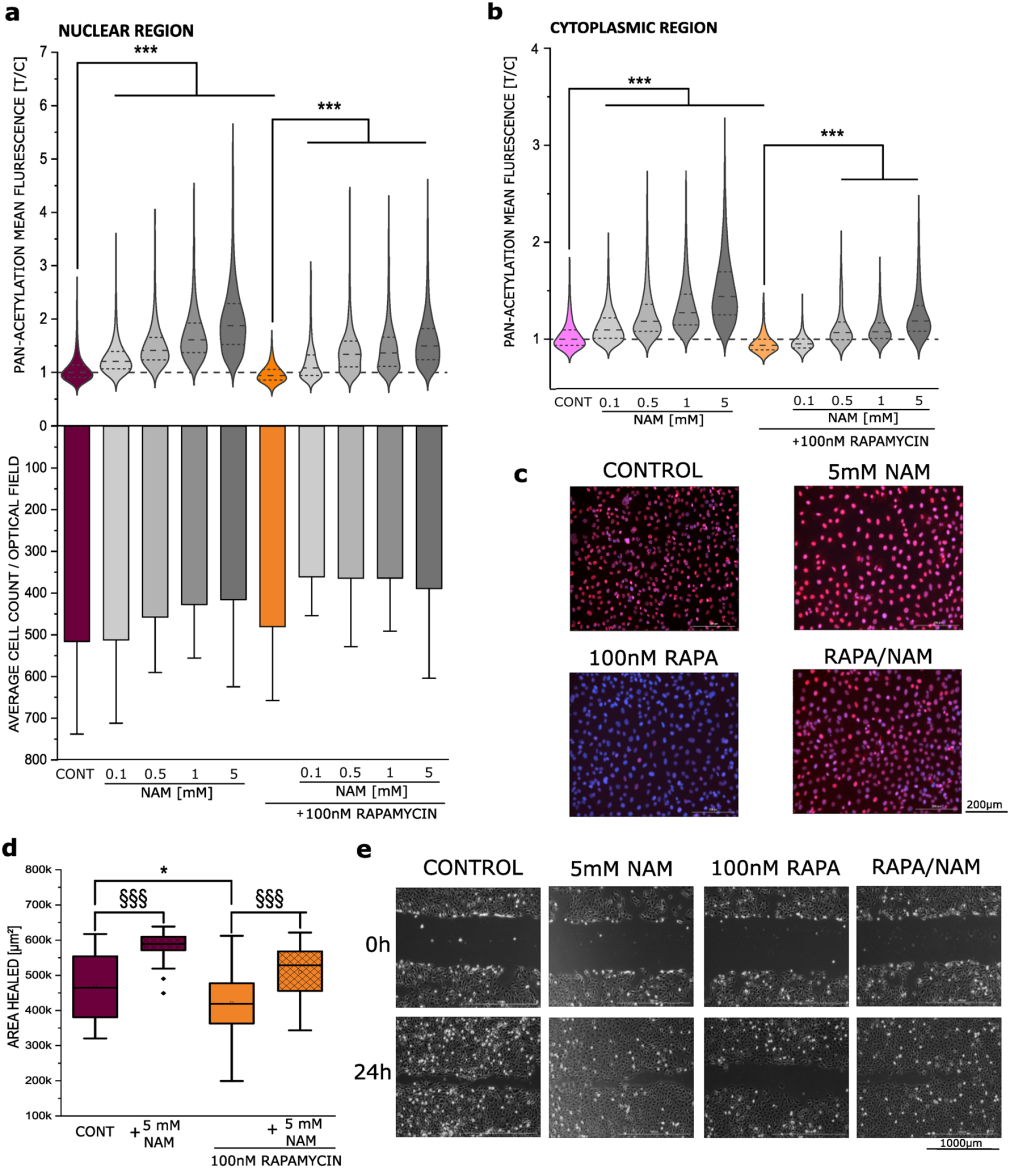
Changes in acetylation and migration following co-incubation with nicotinamide (NAM) and rapamycin (RAPA). **a**, **b** Quantification of acetylation in T24 cells; cells were incubated with increasing NAM concentrations with and without RAPA; results are given as the ratio of mean fluorescence intensity of acetylated lysine of the treatment and the average of the control (*n* = 27 optical fields) statistical significance is shown via Student’s *t*-test: *** (*p* < 0.001); cell count is given as average number of DAPI stained nuclei per quantified image **c** representative images of the experiment; DAPI is shown in blue (nuclei), acetylated lysine in red, (scale bar: 200 µm). **e** Quantification of the gap-closure assay, results are given area healed [µm^2^], calculated from the initial area of the wound minus the area at the same coordinates after 24 h incubation; * indicates a significant difference to controls and § among treatments at the Mann–Whitney Test (*p < 0.05; §§§*p* < 0.001); **e** Representative images at both *t* = 0 and *t* = 24 hours, taken with phase contrast (scale bar: 1000 µm)

Taking as starting point the lack of compensatory effects between autophagy activation and inhibition, alternative molecular pathways appeared to be involved in the regulation of the migratory capacity of T24 cells. Considering the loss of mitochondria triggered by rapamycin, an involvement of cell metabolism appeared plausible. Hence, we postulated that rapamycin could hamper cell biomechanical compliance via a change of intracellular acetylation mediated by sirtuin deacetylase (SIRT1) and change in this way the post-translational modifications of crucial cytoskeletal elements. To explore this hypothesis, we tested the effect of nicotinamide (NAM 0.1–5 mM), a known inhibitor of SIRT1 (Avalos et al. 2005) and we compared it to the effects of 100 nM rapamycin. In line with the described mechanism of action, incubation with NAM increased significantly acetylation levels in a concentration-dependent manner in both the nucleus and cytosol area of T24 cells. As for the previous measurements, rapamycin decreased acetylation compared to control. Additionally, NAM antagonized the reduction in acetylation triggered by rapamycin, starting from the concentration of 0.1 mM (Fig. 6a). Similarly, in the cytoplasmic region acetylation loss induced by rapamycin was restored (> 0.5 mM; Fig. 6b–c).

After describing that NAM could restore acetylation in rapamycin treated cells, we wanted to verify if this could be relevant in the functional response of T24 bladder cells. Migration experiments were performed comparing NAM to rapamycin treatment. In agreement with the results of the acetylation assay, NAM increased migration/gap-closure efficiency in T24 bladder cancer cells (Fig. 6d and e). Importantly, NAM also restored the migration capacity of rapamycin treated cells back to control levels. This result shows that not only could the acetylation itself be restored by SIRT1 inhibition, but also functionally affect cell motility (Fig. 6d–e).

## Discussion

Molecular pathways linking cancer progression and autophagy modulation are tightly connected (Levy et al. 2017; Mulcahy Levy and Thorburn 2020). This is especially relevant in the bladder, where chemical composition of the urine varies according to dietary habits, lifestyle and exposure to xenobiotics, fostering in this way adaptive capacity of bladder cells (Abia et al. 2013; Oesterle et al. 2022). Additionally, pharmacological intervention contributes to the complex environment which confronts this cell type. Crucially for therapy, bladder cancer cells were previously described to take advantage of autophagy activation to become chemoresistant (Li et al. 2019b), thus raising potential concerns about concomitant exposure to autophagy inducing chemicals. Importantly, while cell metabolism is modifiable via physical cues (Romani et al. 2021), rather little is known on the role of autophagy in shaping bladder cells mechanotransduction, which is essential for the adaption to voiding–filling cycles of the organ as well as for metastasis formation. In this study we used in vitro generated fluid shear stress as well as chemical activation of mechanosensitive PIEZO1 channels to recreate in vivo shear stress conditions (Davies et al. 2019). We previously described that incubation with rapamycin modifies the morphometric adaption of T24 cells to shear stress (Del Favero et al. 2021b). Here, in line with previous works (Tuloup-Minguez et al. 2013), we found that this effect is related to a reduction of cell migratory potential (Fig. 1a–b). Considering that starvation induced autophagy (Supplementary Fig. 1) enhanced migration and not reduced it like rapamycin, we considered that the autophagy activator could have acted specifically on cascades governing cell motility. In line, rapamycin incubation was accompanied by a reduction of the translocation efficiency of the mechanosensitive transcription factor KLF2 (Paolini et al. 2021) (Fig. 1c–e). Since the latter was detectable exclusively in presence of physical movement of the extracellular fluids and not following stimulation with PIEZO1 agonists YODA1 (Fig. 1c–d and f), we concluded that cytoskeletal integrity of T24 cells must have been altered by the incubation with rapamycin and not the translocation potential of the transcription factor per se*.* Additionally, despite a previous study in prostate cancer describing an inhibitory effect of KLF2 on migration (Wang et al. 2019), we found decreased nuclear KLF2 following rapamycin incubation. This suggests more likely that KLF2 could reflect the autophagy modulation rather than a role in regulating migration. This would imply increased KLF2 degradation or alteration of cell architecture, which mirrors on the localization of the transcription factor (Wang et al. 2013; Liu et al. 2013). Indeed, the cytoskeleton allows the cells to adapt to external mechanical stress (Matthews et al. 2006; Banerjee et al. 2020). Furthermore autophagy (Hernández-Cáceres et al. 2021) migration (Carlier et al. 2003) but also localization of transcription factors (Itoh et al. 1999) are highly dependent on it. KLF2 physiologically responds to shear stress and previous studies have shown that this competence is lost if the cytoskeleton is impaired (Boon et al. 2010). Therefore we investigated several cytoskeletal proteins and found that rapamycin had a significant effect on the distribution of actin. Additionally, rapamycin reduced integrins and promoted their organization on the cell periphery (Fig. 2c), the latter being essential for migration and invasion of cancer cells, and not only mediating cell adhesion, but also shaping cytoskeletal organization. Taken together these modifications of cytoskeletal function might translate into the inhibition of migration, which we also observed (Figs. 1, 5 and 6) (Hood and Cheresh 2002). Importantly, we could not observe changes in cell membrane morphology or fluidity, which could also account for altered response to shear stress and subsequent loss of cytoskeletal integrity (Fig. 3a and b) (Del Favero et al. 2018). In addition to the effects on the cytoskeleton, we realized that rapamycin triggered a loss of peroxisomes (Fig. 3c and d), which could infer for a reduced capacity of the cell to perform beta-oxidation of fatty acids (Wanders et al. 2016); this in turn, would reduce the generation of NADH from NAD + and contribute to a disbalance of the NAD + /NADH ratio. Previous studies already described the capability of rapamycin (100 nM) to increase the NAD + /NADH ratios in skeletal muscle cells (Zhang et al. 2020). Following this line of interpretation, we investigated mitochondria (Fig. 4), as they contribute most of the cells NADH production, by quantifying mitochondrial import receptor subunit TOM20 located on the outer mitochondrial membrane (Bhagawati et al. 2021) and clustered mitochondria protein homolog (CLUH). The latter has been connected to mitochondrial fission, the splitting of one mitochondria in two daughter mitochondria, a process tightly connected to quality control of mitochondria (Westermann 2010; Yang et al. 2022). Rapamycin decreased TOM20 signal as suggestive of a mitochondrial loss. Increase in CLUH signal points to mitochondrial fission. Taken together, our results describe a signature which is suggestive of increased autophagy seen as mitochondrial and peroxisomal turnover. Our hypothesis at that point was that the loss of peroxisomes and mitochondria via rapamycin induced a change in the NAD + /NADH ratio, which would trigger the activation of sirtuins (Anderson et al. 2017). Sirtuin1 (SIRT1) dependent deacetylation of cortactin has previously been shown to promote branching and polymerisation of actin (Zhang et al. 2009; A et al. 2020). Furthermore, following a recent paper (Lovy et al. 2020) SIRT1 itself can induce further mitochondrial fragmentation via changes in the cortactin-mediated actin polymerisation, plausibly resulting in a self-enhancing loop of reducing mitochondria even further. Following this interpretation would be coherent also with the data described for bafilomycin (Fig. 5d–e) and with the lack of compensatory capacity observed in the combination of bafilomycin with rapamycin (Fig. 5d–g). It was previously demonstrated that bafilomycin can trigger mitochondrial uncoupling (Zhdanov et al. 2012) and accumulation of aged mitochondria upon autophagy deficiency could also account for energetic imbalance and increased glycolysis (Del Favero et al. 2020a). Hence, even with different molecular effectors both autophagy activation and inhibition could potentially account for alteration of mitochondrial bioenergetics, and subsequent sirtuins activation. Together with our results this shows that metabolic competence has the potential to affect cell motility and that increased SIRT1 dependent deacetylation could be a key factor in the inhibition of cell migration. Most importantly, it also infers for a great potential of the metabolic intervention in shaping the outcome of pharmacological treatment or exposure to chemicals. To confirm our hypothesis, we combined rapamycin treatment with the established SIRT1 inhibitor nicotinamide (NAM). NAM reverted rapamycin-induced acetylation signature and functional effect on migration (Fig. 6). The restoration of acetylation capabilities strongly supports the role of SIRT1 mediated deacetylation, whereas the increase in migration further strengthens the dependency between cell motility and overall acetylation status inside the cell. NAM is already considered as a potential treatment for metabolic disorders. Most metabolic diseases feature a similar dysregulation of their NAD + /NADH homeostasis, such as Diabetes Mellitus (Maiese 2020) or Danon (Del Favero et al. 2020a) and Leigh (Lee et al. 2019) disease. In line with our results, a leading cause of this imbalance is mitochondrial dysfunction. NAM can be used to support metabolic and mitophagic pathways, because of its protective function on mitochondria, as well as its role as a precursor of NAD +  (Maiese 2020). In Leigh disease nicotinamide mononucleotide NMN, another endogenous precursor of NAD + , was used to normalize NAD + /NADH ratio (Lee et al. 2019). In bladder cancer, NAM is used in combination with carbogen, to restore cellular respiration, overcoming resistance induced by hypoxic conditions during radiotherapy (Hoskin et al. 2010). However as shown in our results (Fig. 6) NAM increases migration of bladder cancer cells significantly. Considering that autophagy modulators are considered an option to support therapeutic intervention in bladder cancer (Li et al. 2019a) and that some treatments induced autophagy, it is crucial to explore multiple facets of this intervention. While NAM clearly is a great therapeutic option as a chemosensitizer, its use might under certain circumstances even increase the metastatic potential of the tumor; however, if used just before the radiotherapy, as described (Hoskin et al. 2010), detrimental effects should be insignificant. Importantly, the data collected with our model, despite the obvious limitation of an in vitro setup, clearly bring forth that mechanotransduction is a valuable readout to assess the molecular mechanisms of pharmaco-toxicological response of bladder cells. This has a great potential, since every cell possesses multiple mechanosensors to detect physical stimuli, such as mechanically activated ion channels, e.g., PIEZO1/2) (Ranade et al. 2014; Saotome et al. 2018) or via the cytoskeleton itself (e.g., Caveolin (Yang et al. 2016; Moreno-Vicente et al. 2018), Integrins (Ross et al. 2013), Filamin A (Razinia et al. 2012; Ehrlicher et al. 2011). This is valuable in light of the increasing importance of physical stimuli in cancer pathophysiology, including the connection between shear stress and metastasis (Yang et al. 2016) as well as resistance to chemotherapy (Ip et al. 2016). For example, it was already demonstrated that low shear stress (LSS) induced cell motility and metastasis via Caveolin-1 and PI3K/Akt/mTOR-mediated pathways (Yang et al. 2016; Xiong et al. 2017). This on the one side contributes to shed light on the complex crosstalk between biochemical and biomechanical pathways occurring in the cells and, in addition, promises to open new perspectives for the development of novel therapeutic approaches.

## Conclusion

Overall, the data collected in this study suggest that an increased turnover of mitochondria could result in sirtuin mediated lysine deacetylation. Following the changes in this crucial PTM, a signature of cytoskeletal impairment was observed, which clearly reflected on cell mechanotransduction. Rapamycin successfully hampered the cells motility and adaptiveness to shear stress. However, metabolic intervention with NAM readily reverted this response, and proved more efficient than simply autophagy inhibition. Exemplified in the study of bladder cancer cells, this shows the great potential of metabolic modulation in health and disease and contributes to the comprehension of the impressive adaptive capacity of bladder cancer cells.

## Supplementary Information

Below is the link to the electronic supplementary material.

Supplementary file1 (DOCX 248 KB)
