# Supplementary material for: Activation of autophagy triggers mitochondrial loss and changes acetylation profile relevant for mechanotransduction in bladder cancer cells
Source: Arch Toxicol. 2022 Oct 10;97(1):217–33. doi: 10.1007/s00204-022-03375-2 (PMC9816236; doi:10.1007/s00204-022-03375-2)
Supplement: Supplementary file 1 — Supplementary file1 (DOCX 248 KB) [file 204_2022_3375_MOESM1_ESM.docx]

**Activation of autophagy triggers mitochondrial loss and changes acetylation profile relevant for mechanotransduction in bladder cancer cells**

Maximilian Jobst^1^, Endre Kiss^2^, Christopher Gerner^3,4^, Doris Marko^1^, Giorgia Del Favero^1,2^*

1 Department of Food Chemistry and Toxicology, University of Vienna Faculty of Chemistry, Währingerstr. 38-40, 1090 Vienna

2 Core Facility Multimodal, Imaging, University of Vienna Faculty of Chemistry, Währingerstr. 38-40, 1090 Vienna

3 Department of Analytical Chemistry, University of Vienna Faculty of Chemistry, Währingerstr. 38-40, 1090 Vienna

4 Joint Metabolome Facility, University of Vienna and Medical University of Vienna, Vienna

* Correspondence: G. Del Favero, giorgia.del.favero@univie.ac.at

SUPPLEMENTARY DATA:

Supplementary figure 1:


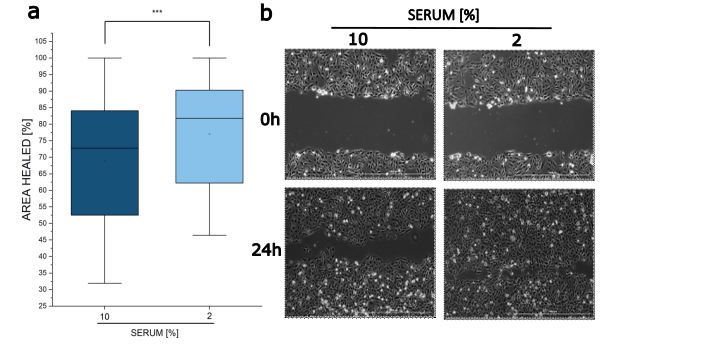


Migration experiments after 24h of serum starvation (2%). Cell migration as healed area in percentage following serum starvation (a), significance is shown via students t-test: *** (p < 0.001). Data results from the evaluation of n=12 optical fields from 4 independent cell preparations. Representative images at both t=0 (left) and t= 24 (right) hours (b), taken with phase contrast (scale bar: 1000 µm).

Supplementary figure 2


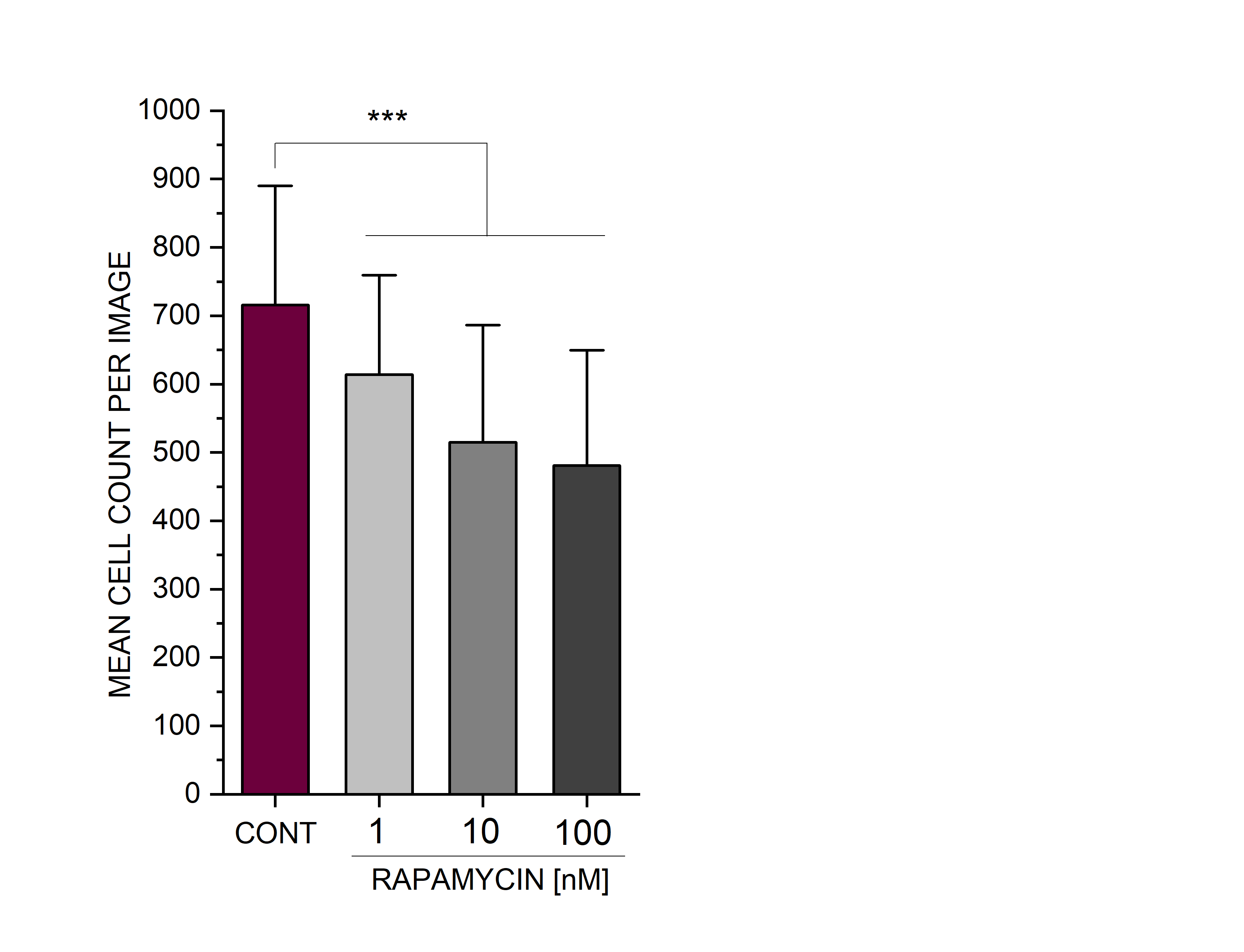


Mean cell count per image, detected as DAPI stained nuclei, significance is shown via students t-test: *** (p < 0.001). Data results from the evaluation of n=27 optical fields from 3 independent cell preparations.
